# Is There Still Room for Cancer Vaccines at the Era of Checkpoint Inhibitors

**DOI:** 10.3390/vaccines4040037

**Published:** 2016-11-03

**Authors:** Soumaya Karaki, Marie Anson, Thi Tran, Delphine Giusti, Charlotte Blanc, Stephane Oudard, Eric Tartour

**Affiliations:** 1 INSERM U970, Université Paris Descartes, Sorbonne Paris-Cité, 75015 Paris, France; soumaya.karaki@inserm.fr (S.K.); marie.anson@inserm.fr (M.A.); thi.tran@inserm.fr (T.T.); Charlotte.blanc@gmail.com (C.B.); stephane.oudard@aphp.fr (S.O.); 2 Hôpital Européen Georges Pompidou, AP-HP, Service d’Immunologie Biologique, 75015 Paris, France; delphine.giusti@aphp.fr; 3 Department of Oncology, Hôpital Européen Georges Pompidou, 75015 Paris, France; 4 Equipe Labellisée Ligue contre le Cancer, 75015 Paris, France

**Keywords:** cancer vaccine, checkpoint inhibitors, immunotherapy, combination therapy, PD-1, PD-L1, CTLA-4, CD40, OX40

## Abstract

Checkpoint inhibitor (CPI) blockade is considered to be a revolution in cancer therapy, although most patients (70%–80%) remain resistant to this therapy. It has been hypothesized that only tumors with high mutation rates generate a natural antitumor T cell response, which could be revigorated by this therapy. In patients with no pre-existing antitumor T cells, a vaccine-induced T cell response is a rational option to counteract clinical resistance. This hypothesis has been validated in preclinical models using various cancer vaccines combined with inhibitory pathway blockade (PD-1-PDL1-2, CTLA-4-CD80-CD86). Enhanced T cell infiltration of various tumors has been demonstrated following this combination therapy. The timing of this combination appears to be critical to the success of this therapy and multiple combinations of immunomodulating antibodies (CPI antagonists or costimulatory pathway agonists) have reinforced the synergy with cancer vaccines. Only limited results are available in humans and this combined approach has yet to be validated. Comprehensive monitoring of the regulation of CPI and costimulatory molecules after administration of immunomodulatory antibodies (anti-PD1/PD-L1, anti-CTLA-4, anti-OX40, etc.) and cancer vaccines should help to guide the selection of the best combination and timing of this therapy.

## 1. Introduction

Recognition of an HLA-peptide complex on antigen-presenting cells (APC) and delivery of a second signal by the interaction of positive costimulatory molecules with their ligands expressed on T cells are both required for priming of T cells. Some receptors are already present on naïve T cells (CD28, CD27), but other costimulatory molecules (4.1BB, OX40, CD40L) will be expressed during the T cell activation process, which, following their interaction with their ligands (4-1BBL, OX-40L, CD40), modulate the quality and intensity of T cell response in terms of polarization and differentiation in memory T cell subpopulations. To regulate this activation process, inhibitory receptors (CTLA-4, PD-1, LAG-3, Tim-3, etc.) are also induced after activation to allow the return of T cells to a resting state. In cancer, due to the chronic persistence of antigens and the general inefficacy of the antitumor response to eradicate the tumor, chronic T cell activation results in sustained expression of inhibitory receptors and dysfunctional or exhausted T cell status. Alison′s group was the first to hypothesize that blocking these inhibitory receptors could reverse the poor functionality of intratumoral T cells. In 2010, a phase 3 clinical trial demonstrated that anti-CTLA-4 mAb improved overall survival (OS) in previously treated metastatic melanoma patients and, in 2011, it was shown that ipilimumab (Yervoy^®^) combined with standard chemotherapy also improved clinical outcome in previously untreated metastatic melanoma patients compared to chemotherapy alone [1]. In 2011, Topalian et al. reported the efficacy of an anti-PD-1 antibody in metastatic melanoma, renal cell carcinoma and lung cancer patients [2]. Four years after this pioneer work, this novel therapeutic class of molecules targeting inhibitory receptors clearly represents a breakthrough in the treatment of cancer. After various phase 3 clinical trials, the anti-PD-1 mAb, nivolumab (Opdivo^®^) has been approved for the treatment of advanced melanoma, metastatic lung cancer, metastatic renal cancer and Hodgkin′s lymphoma. At the present time, pembrolizumab (Keytruda^®^), another anti-PD-1 mAb, has been approved by the FDA for the treatment of metastatic melanoma, metastatic non-small cell lung cancer, and recurrent or metastatic head and neck squamous cell carcinoma. Recently, the anti-PD-L1 mAb, atezolizumab (Tecentriq^®^) has been conditionally approved for metastatic urothelial tumors. Finally, the FDA has granted accelerated approval to nivolumab (Opdivo^®^) in combination with ipilimumab (Yervoy^®^) for the treatment of patients with BRAF V600 wild-type, unresectable or metastatic melanoma. Despite these dramatic and rapid clinical successes, it has now been established that these various immunomodulators provide clinical benefit in only 25%–30% of treated cancer patients. In addition, various cancers (prostate cancer, colorectal cancer without microsatellite instability (MSI), sarcomas, pancreatic cancer, non-triple-negative breast cancer, etc.) present primary resistance to these immunomodulators.

Cancer vaccines represent another immunotherapeutic approach based on stimulation of the antitumor immune response after immunization with a defined nominal tumor antigen or modified tumor cells. The clinical benefit of these cancer vaccines has been disappointing in most studies. However, several recent clinical trials have reported encouraging results for this immunotherapy. In a phase 3 clinical trial, Sipuleucel-T, a cancer vaccine based on pulsing of autologous APC with a chimeric protein composed of phosphatase acid prostatic protein fused to GM-CSF, improved the median overall survival by 4 months in metastatic castration-resistant prostate cancer patients [3]. However, the difficulty of the manufacturing process and the benefit-cost ratio prevented this product from becoming a standard of care for these patients. Since HPV infection is associated with 5%–7% of cancer (cervical cancer, oropharyngeal cancer, anal cancer, etc.), various therapeutic HPV vaccines have been developed with encouraging results, especially in patients with preneoplastic lesions [4,5,6,7,8].

Two novel approaches have been investigated to improve the efficacy of cancer vaccines: (i) induction of CD8^+^T cells in the blood has been considered to be a good surrogate marker of the potency of a cancer vaccine. Nevertheless, preclinical results tend to show that the induction of a subpopulation of resident memory T cells that persist in the tissue and do not recirculate may be a prerequisite for vaccine efficacy [9,10,11]; (ii) Next-generation sequencing now rapidly identifies the mutanome of a given tumor and an algorithm can be used to predict potential neoepitopes that could constitute attractive tumor antigens, as this mutated antigen is specific to the tumor and recognition of this antigen is not controled byvarious central thymic and peripheral (regulatory T cells (Treg), MDSC, etc.) tolerance mechanisms associated with self-antigens expressed by both normal and tumor cells. Early clinical trials have shown that these personalized cancer vaccines based on each patient’s mutanome are immunogenic and can provide clinical benefit [12,13].

It is noteworthy that cancer vaccines have been essentially evaluated in metastatic disease associated with more pronounced immunosuppression, which may explain clinical failure, while positive clinical signals were observed mainly in preneoplastic lesions. The clinical setting in which a cancer vaccine is tested may be of utmost importance, as the same vaccine can induce a clinical response on early-stage vulvar disease, but not on late-stage cancer [4,14]. Although the Her2-Neu cancer vaccine was mainly proposed in patients expressing high levels of Her2/neu in their tumors [15], we have recently demonstrated that an Her2/neu targeting cancer vaccine is more efficient in low Her2/neu expressing tumors, as Her2 downregulates HLA-class I expression [16].

The current view, especially in metastatic disease, is that cancer vaccines must be combined with molecules able to reverse immunosuppression present in the tumor microenvironment and/or promote the recruitment of antitumor effector cells in situ [17,18]. In this context, anti-angiogenic molecules, and certain forms of chemotherapy and radiotherapy have been shown to potentiate cancer vaccines [19,20,21,22,23,24,25].

The combination of cancer vaccines with checkpoint inhibitor blockade represents another option to improve clinical benefit and a way to reverse resistance to immunomodulators [26]. Although the preliminary clinical results appear to be very promising, other strategies combining checkpoint inhibitor blockade with other molecules such as IDO inhibitors, which could also improve antitumor immune response, will not be discussed in this review [27,28,29].

## 2. Rationale for the Combination of Cancer Vaccines AND Checkpoint Inhibitor Blockade

As expected from the known physiological steps of T cell activation, we and other teams have shown an induction of inhibitory receptors after vaccination during the late stage of activation both in preclinical models and in humans [30,31]. Blocking these inhibitory pathways should lead to amplification of the T cell-mediated immune response considered to be the main effector of cancer vaccines [32]. IFNγ production by antitumor-specific T cells could also upregulate PD-L1 on tumor cells, as a resistance mechanism to adaptive immunity, thereby promoting PD-L1-PD-1 blockade after vaccination [33,34,35]. 

Treg cells are increased in cancer patients. In various models, it has been shown that these cells can inhibit both the priming and the function of antitumor effector T cells after vaccine administration [36,37,38]. Some inhibitory (CTLA-4, LAG-3, PD-1, etc.) and agonist receptors (GITR, OX40) are expressed on Treg cells, but also on effector T cells [39]. Anti-CTLA-4 mAb have been shown to deplete Treg via Fc receptors in murine models and some authors have claimed that the antitumor activity of these antibodies is mediated via this action on Treg cells [40,41]. Agonist antibodies directed against OX40 may have a dual role, inhibiting Treg cell suppression, while enhancing effector T cell functions [39,42,43]. Agonist anti-GITRs have also been shown to make effector T cells more resistant to the inhibition induced by Treg cells [44]. In view of their actions on the modulation of Treg activity, the combination of cancer vaccines with costimulatory molecule agonists or checkpoint inhibitor antagonists would therefore be justified.

Conversely, the clinical efficacy of antibodies blocking certain inhibitory pathways such as PD-1-PD-L1 requires the pre-existence of antitumor CD8^+^ T cells at the tumor site [45]. These antibodies appear to reactivate disabled intratumoral T cells. Since it has been shown that about 70% of cancers are not significantly infiltrated by CD8^+^ T cells [46], cancer vaccines could allow the priming and intratumoral recruitment of these cells and could transform a “non-inflamed” non-permissive tumor resistant to checkpoint inhibitor blockade into a sensitive “inflamed” tumor [47]. Induction of a CD8^+^ T cell response after cancer vaccine administration [23,48] and, in some cases, recruitment of these T cells in the tumor have been demonstrated in humans [49].

## 3. Combination of Cancer Vaccines and Modulation of Costimulatory Pathways in Preclinical Models (Table 1)

### 3.1. Inhibitory Pathway (PD-1/PD-L1-PD-L2, CTLA-4/CD80-CD86) Blockade Increases the Efficacy of Various Types of Cancer Vaccines

In animal models, combined therapies with CPI and various types of cancer vaccines have demonstrated enhanced activation of vaccine-induced tumor-specific T cells and synergistic antitumor effects [50,51]. Several different vaccination approaches have been explored to enhance the efficacy of immune checkpoint blocking antibodies, including simple vaccine preparations consisting of specific peptides and proteins, as well as more complex strategies, such as engineered cellular vaccines, DC vaccines, and virus-vectored vaccines. 

#### 3.1.1. Peptide Vaccines

Although synthetic peptides are probably one of the simplest types of vaccines to prepare and characterize, these compounds are usually not very immunogenic.

Combined therapy with a peptide vaccine derived from glypican-3 and expressed in hepatocellular carcinoma and anti-PD-1 mAb synergistically suppressed tumor growth. In a tumor-bearing mouse model, PD-1 blockade increased the number of peptide-specific tumor-infiltrating T cells (TILs) and decreased the expression of inhibitory receptors on TILs [74]. In line with these results, the combination of a survivin peptide with anti-CTLA-4 was associated with the generation of survivin-specific T cells and long-term survival of mice in a therapeutic setting, but not in a prophylactic experiment (i.e., before tumor challenge) [75].

Vaccination with a synthetic peptide corresponding to an immunodominant self CD8 epitope derived from tyrosinase-related protein-2 (TRP2) administered with CpG-ODN adjuvant and followed by systemic injection of anti-CTLA-4 antibodies increased the survival of mice against the poorly immunogenic B16 melanoma. Interestingly, although this combination therapy was effective when administered to tumor-bearing mice (therapeutic protocol), it had no significant effect when applied in the prophylactic setting [57].

#### 3.1.2. Live Vectors

Several studies have investigated virus-vectored vaccines (vaccinia, fowlpox, adenovirus, and lentivirus) or other live vectors, as a means to enhance the immune response to a specific antigen in the context of immunomodulatory antibody therapy.

The combination of CTLA-4 blockade with a modified vaccinia Ankara-expressing murine p53 has been reported to act synergistically to reject palpable Meth A, 11A-1 and MC-38 tumors. In vivo, Ab depletion confirmed that the antitumor effect was primarily CD8-dependent and, to a lesser extent, CD4-dependent. CpG enhanced the efficacy of CTLA-4 blockade [58,62].

Similarly, a combination of the recombinant vaccinia vector carrying the genes for CEA, B7.1, ICAM-1, and LFA-3 (rV-CEATRICOM) and recombinant fowlpox-boosted vaccines with systemic CTLA-4 blockade led to enhanced antitumor immunity [63].

Lastly, prophylactic vaccination with a highly attenuated *Trypanosoma cruzi* strain expressing a cancer-testis antigen, NY-ESO-1 (CL-14-NY-ESO-1) combined with anti-CTLA-4 was highly effective in controlling the development of an established melanoma [76].

Vaccination with recombinant lentivirus encoding tumor antigen combined with modulation of the PD-1-PD-L1 pathway by PD-1 or PD-L1 blocking antibodies enhanced vaccine efficacy and improved antitumor immunity [77].

#### 3.1.3. Cellular Vaccines

Preclinical studies have reported that the combination of CTLA-4 blockade and a vaccine composed of granulocyte–macrophage colony-stimulating factor (GM-CSF)-expressing tumor cells (GVAX) resulted in regression of parental mammary carcinoma or melanoma or prostate cancer cells [52,54], while each treatment alone was ineffective. CD8^+^T cells were involved in the efficacy of combined therapy. A similar effect the improvement of the GVAX vaccine was observed when in combination with PD-1 blockade [78].

In the poorly immunogenic B16 melanoma model, vaccination with TEGVAX (GM-CSF-secreting tumor cell vaccine combined with TLR agonists) was only able to slow but not eliminate tumors and anti-PD-1 antibody alone had minimal activity. Significant tumor regression was observed when TEGVAX was administered concurrently with anti-PD-1 antibody [69].

Dendritic cells (DC) are considered to be the only APC able to prime naïve T cells, making them attractive candidates to be included in the design of cancer vaccines.

In the EL4 mouse thymoma model, it has been reported that neither DC-vaccination nor anti-CTLA-4 therapy alone is able to influence tumor growth, whereas combined therapy induced effective tumor rejection or growth inhibition [59,60,79]. In line with these results, blockade of PD-L1 signaling during DC vaccination showed better therapeutic effects than classic DC vaccination by preventing tumor growth and prolonging survival times in a breast tumor-bearing hu-SCID model [68].

#### 3.1.4. Inert Vectors Targeting Dendritic Cells

To take advantage of the potency of DC to elicit antitumor immune responses, while avoiding the time-consuming process of DC generation, we and other groups have developed non live vectors that are able to preferentially deliver antigen to DC [80,81,82,83,84].

In a preclinical model of PD-L1-expressing HPV(+) tumors, we demonstrated that administration of anti-PD-L1 in the absence of cancer vaccine was inefficient to control growth of the tumor, which was not infiltrated by CD8^+^T cells. Immunization of mice with a vaccine based on the B subunit of Shiga toxin, which binds the Gb_3_ receptor preferentially expressed on DC, coupled to the E7 protein derived from HPV was partially efficient to inhibit tumor growth. A synergy was observed when the vaccine was combined with anti-PD-L1 mAb [31].

DEC 205 is a lectin preferentially expressed on lymphoid DC in mice. Combination of an anti-DEC-205 (dendritic and epithelial cells, 205 kDa)-HER2 (human epidermal growth factor receptor 2) vaccine with a dual agonist antibody directed against OX40 and an antagonist antibody directed against CTLA-4 significantly improved survival in a mammary carcinoma model. This combined therapy was associated with extensive tumor destruction and T-cell infiltration in the tumor [67].

#### 3.1.5. DNA Vaccines

The antitumor activity of a DNA vaccine encoding the cancer-testis antigen SSX2, modified to encode altered epitopes with increased MHC class I affinity, can be increased when combined with PD-1- or PD-L1-blocking antibodies [85].

SCIB2, an antibody DNA vaccine encoding NY-ESO-1 epitopes, induced potent antitumor immunity, which was further enhanced by CTLA-4 or PD-1 blockade [86].

### 3.2. Synergy between Cancer Vaccines and Checkpoint Inhibitor Blockade Extends Beyond CTLA-4 and PD-1 Pathway Inhibition

In addition to CTLA-4 and PD-1, several other inhibitory receptors, such as LAG-3 and TIM-3, have been shown to be expressed during later stages of T cell activation.

Combining LAG-3 blockade with specific antitumor vaccination based on recombinant vaccinia virus resulted in a significant increase in activated intratumoral CD8^+^ T cells in the tumor. A major component of this effect was CD4-independent and required LAG-3 expression by CD8^+^ T cells [87].

In a model of irradiated B16 melanoma cells expressing the flt3 ligand gene (FVAX), Baghdadi et al. showed that treatment with anti-Tim-3 mAb increased the numbers and activity of tumor-infiltrating natural killer (NK), whereas anti-Tim-4 mAb administration resulted in an increase of CD8^+^ T cell functions. When administered together in combination with the vaccine, the two blocking antibodies significantly suppressed tumor growth [88].

A novel immunoglobulin (Ig) superfamily ligand, designated V-domain Ig suppressor of T cell activation (VISTA), which acts as a checkpoint inhibitor in cancer models, has been recently discovered [89,90]. In the hematopoietic compartment, VISTA is expressed on tumor-infiltrating myeloid cells. LeMercier et al. examined the use of an anti-VISTA mAb in combination with a tumor vaccine and found that VISTA blockade impaired tumor growth in a B16-BL6 tumor model [73].

### 3.3. Checkpoint Inhibitor Blockade is Not Limited to the Use of Antibodies

Soluble molecules inhibiting the interaction of inhibitory receptors with their ligands have been developed.

Song et al. also reported that administration of soluble PD-1 DNA (which should result in soluble PD-1 binding to PD-L1, thereby blocking PD-L1 ligation of T cells expressing PD-1) with either a human papilloma virus-16 E7 DNA vaccine or adenovirus-based vaccine significantly increased the magnitude of E7-specific CD8^+^ T-cell responses and antitumor efficacy against E7-expressing tumors with no difference in efficacy between the two vaccines [91].

Activation of the BTLA-HVEM co-inhibitory signaling pathway impairs antitumor immunity. Blockade of this pathway by HSV-1 gD, a protein derived from herpes simplex virus which binds to HVEM, combined with active immunization resulted in sustained tumor regression in a transgenic mouse model with slowly progressing thyroid gland tumors [72]. Another study showed that, in the presence of gD, the CD8^+^T cells induced after vaccination with a replication defective adenoviral vector exhibited reduced expression of 2B4, LAG-3 and PD-1 a hallmark of a less exhausted phenotype [92].

A soluble form of BTLA encoded by recombinant adeno-associated virus (AAV) or a DNA plasmid, in combination with an HSP70 vaccine generated a potent antitumor response in the melanoma lung metastasis model in B6 mice and in a lung epithelial tumor [93,94].

### 3.4. Agonist Antibodies Directed Against Costimulatory Molecules are also Synergistic with Cancer Vaccines

Apart from blockade of inhibitory molecules expressed on exhausted T cells, agonist antibodies have also been tested in order to activate costimulatory receptors to improve the efficacy of antitumor vaccines [95,96,97]. Agonist antibodies act by increasing the frequency and function of APCs and T cells [98].

Members of the tumor necrosis factor receptor (TNFR) (CD40, OX40 (CD134), 4-1BB (CD137), GITR (CD357), etc.) family have emerged as key costimulatory molecules for enhanced effector function and memory cell development. Expressed on activated T and B lymphocytes and APCs, ligation of some of these receptors has been shown to promote cell division and survival, and facilitate cell differentiation, maturation of APCs, and direct signaling in T cells [99,100].

Anti-CD40 mAb amplified T cell response via activation of DC or directly on T cells. Anti-CD40 mAb synergized with a peptide vaccine to promote T cell priming [101]. In other studies, immunization of mice with anti-CD40 mAb chemically coupled to tumor idiotype resulted in significantly retarded tumor growth [102]. The efficacy of anti-CD40 mAb could be further enhanced by the concurrent delivery of TLR3 or TLR4 or TLR9 ligand adjuvants with the cancer vaccine [102,103,104].

T cell costimulation via OX40 is known to increase CD4^+^ T cell expansion and effector function and enhances the development of T cell memory. Murata et al. have shown that the combination of GM-CSF whole cell vaccination with agonist anti-OX40 mAb effectively induced a durable antigen-specific CD8^+^ T cell response despite established immune tolerance to the target antigen. Moreover, OX40-expression was upregulated on both CD4^+^ and CD8^+^ T cells shortly after administration of the GM-CSF expressing vaccine, highlighting the increased efficacy of OX40 costimulation when combined with a GM-CSF-secreting vaccine [105]. Agonist OX40 antibodies also enhanced the CD4^+^ and CD8^+^ T cell response generated by a DC-based vaccine [106].

Co-administration of an E6-E7 peptide derived from HPV16 with a 4-1BB agonist antibody promoted durable regression of established genital TC1 tumors expressing E6-E7. In contrast, combining this E6-E7 peptide vaccine with checkpoint blockade produced only a modest benefit [97]. Compared to other therapies tested, this vaccine and anti-41BB combination promoted the highest CD8^+^ versus regulatory Foxp3^+^ T cell ratio [97].

### 3.5. Multiple Targeting of Costimulatory or Inhibitory Receptors Dramatically Improved the Efficiency of Cancer Vaccines

Since T cells express multiple inhibitory and costimulatory receptors, various groups hypothesized that the efficacy of cancer vaccines could be enhanced when simultaneously combined with blockade of inhibitory receptors and activation of costimulatory pathways.

#### 3.5.1. Cancer Vaccines Combined with Multiple Inhibitory Receptor Blockade

Genomic and functional signatures from T cells exposed to checkpoint blockade have revealed that CTLA-4 inhibition elicits a “proliferative signature”, while PD-1 blockade modulates cytolytic function on T cells justifying their association [107]. Indeed, combination of CTLA-4 and PD-1 blockade is more effective than either modality alone in promoting the rejection of various murine tumors secreting GM-CSF or Flt-3 [50,64] or other vaccines [66].

#### 3.5.2. Cancer Vaccines Combined with Agonist mAb Against Costimulatory Molecules and Antagonist mAb Against Checkpoint Inhibitors

Various combinations of agonist mAb and antagonist mAb directed against costimulatory and inhibitory receptors have been tested in combination with cancer vaccines.

Anti-CTLA-4 mAb and anti-CD40 mAb administered with liposome-encapsulated peptide vaccine or an adenoviral vector encoding melanoma antigen induced effective tumor-specific CD8^+^ T cells with no detrimental side effects [108,109]. Vaccination with recombinant adenovirus encoding melanoma antigen delayed tumor growth in 30%–40% of mice, while vaccination in combination with anti-CD40 and anti-CTLA-4 mAbs induced complete response [108]. Animals treated with anti-CTLA-4 mAb and anti-OX40 mAb and vaccinated with anti-DEC-205 (dendritic and epithelial cells, 205 kDa)-HER2 (human epidermal growth factor receptor 2) had significantly improved survival in a mammary carcinoma model [67].

Similar results were observed when mice were firstly vaccinated followed by blockade of the PD-1 pathway combined with anti-CD40 or anti-4-1BB [61,65,68,110].

## 4. Mechanisms Mediating the Efficacy of Cancer Vaccines Combined with Inhibitory Pathway Blockade or Costimulatory Pathway Activation

**Figure 1:**
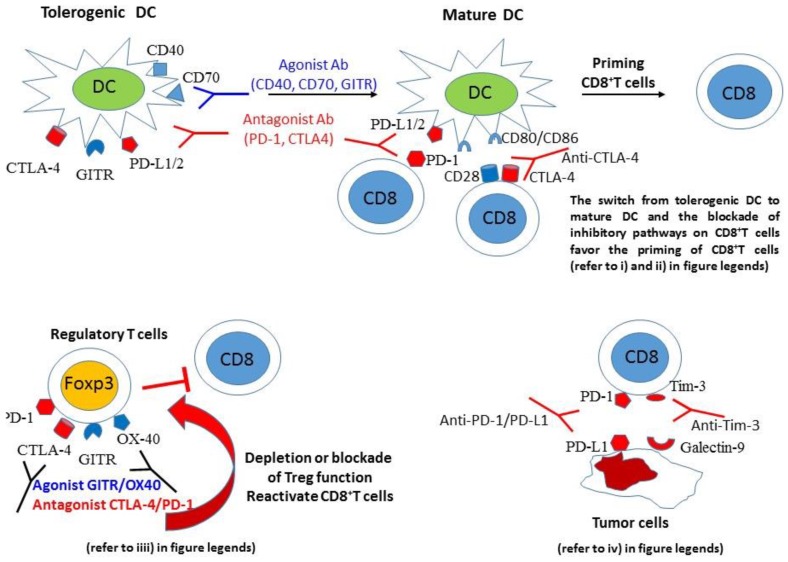
Mechanisms of action of blockade of inhibitory pathways or activation of costimulatory pathways to promote CD8^+^T cell priming after vaccination. Antagonist antibodies targeting checkpoint inhibitors may act at different steps during T cell priming or the antitumor activities of CD8^+^T cells: (i) Blocking CTLA-4, GITR or PD-L1/L2 or activating CD40, CD70 switches dendritic cells from tolerogenic to mature professional antigen-presenting cells that are efficient for CD8^+^T cell priming; (ii) Inhibition of the CTLA-4 and CD80-CD86 interaction promotes the CD28/CD80-CD86 costimulatory pathway; (iii) Inhibition of Treg via targeting of CTLA-4, PD-1, OX40, GITR alleviates the suppressor activity of these cells on effector CD8^+^T cells; (iv) Blockade of the interaction of PD-1/PD-L1 or Tim-3.

Various mechanisms have been implicated in the enhancement of cancer vaccine efficacy secondary to manipulation of costimulatory or inhibitory pathways (Figure 1).

In mice, most of these combinations increased proliferation of antigen-specific effector CD8^+^ and CD4^+^ T cells, release of T cell-derived cytokines and upregulation of key signaling molecules critical for T cell function resulting in enhanced intratumoral infiltration of activated antitumor T cells [31,50,64,67,97]. This observation is consistent with reversal of the disability of T cells due to blockade of inhibitory interactions and activation of their costimulatory pathways.

Various antagonist antibodies directed against CTLA-4 or PD-1 or agonist antibodies directed against OX40 and GITR also modulate Treg cells.

It has been clearly established in murine models that anti-CTLA-4 depleted Treg cells via an Fc gamma receptor-dependent mechanism [40,41]. Nevertheless, the combination of low-dose cyclophosphamide or anti-CD25 to deplete Treg cells and anti-CTLA-4 was more effective than anti-CTLA-4 alone, suggesting that CTLA-4 blockade does not completely mitigate Treg function [55,111,112]. In cancer patients, ipilimumab had no clear impact [113,114] or decreased [115] and sometimes expanded Foxp3^+^CD4^+^ Treg in a dose-dependent manner [116]. However, as Foxp3 is also a marker of activated T cells in humans, the precise modulation of Treg cells in humans by ipilimumab has yet to be defined. Kavanagh et al. observed that CTLA-4 blockade expanded Treg at low doses, but expanded effector T cells only at high doses [116]. Some studies have reported that anti-CTLA-4 or anti-GITR made effector T cells resistant to the inhibitory effect of Treg [44,117,118].

PD-1 signaling in tumors is required to both suppress effector T cells and maintain tumor Tregs and PD-1/PD-L1 pathway blockade enhances tumor inhibition by increasing effector T cell activity, while attenuating Treg cell suppression [50]. However, as also described with anti-CTLA-4, anti-CD25 synergized with anti-PD-1 administered in combination with vaccine in the induction of a strong antitumor immune response and enhanced survival in mice, suggesting that PD-1-PD-L1 pathway blockade did not completely abrogate Treg functions [70]. Increased Treg cells have been observed after PD-1 pathway blockade, especially in non-responding melanoma patients, suggesting that it could constitute an escape mechanism to immunotherapy [119].

When combined with an alphavirus replicon particle-based vaccine encoding the TRP2 melanoma antigen, anti-GITR agonist antibody induced significantly enhanced therapeutic efficacy compared to anti–CTLA-4 mAb plus TRP2 vaccine. This efficacy was associated with decreased infiltration of Tregs and recruitment of phagocytes to the tumor site [120]. Other studies have also shown that OX-40 ligation suppressed Foxp3^+^ Treg [121].

As expected, an increase in the ratio of effector T cells to Treg cells has often been reported during this combined therapy [50].

Other mechanisms that could also explain the antitumor efficacy of the combination of cancer vaccine with blockade of checkpoint inhibitors, especially anti-CTLA-4, include the decreased frequency of myeloid-derived suppressor cells [64,122].

Some agonist antibodies, such as anti-CD40 or anti-CD27, could also directly activate APC or CD8^+^ T cells and bypass the CD4 help normally required for vaccine efficacy [71,123]. Silencing CTLA-4 or GITR on DC enhanced the induction of antitumor CTLs in response to DC transfected with mRNAs encoding either melanoma or breast cancer antigens [124]. Conversely, the immune stimulatory capacity of DC can be optimized by coelectroporation of mRNA encoding CD40 ligand, CD70 and constitutively activated TLR4 [125,126].

Anti-CTLA-4 therapy combined with a TRP2-based vaccine could also increase humoral response with broader specificities compared to the vaccine alone [120]. Prostate cancer patients treated by GVAX and ipilimumab presented antibody responses to filamin B and PSMA (antigens expressed by GVAX), but, in the absence of a control arm, it is difficult to prove the role of ipilimumab in this effect [127].

## 5. Combinations of Cancer Vaccine and Modulation of Inhibitory Pathways in Humans

Ipilimumab combined with tumor antigen peptide vaccine was not associated with any additional clinical benefit in melanoma patients [114,128,129]. Anti-CTLA-4 also does not appear to affect the magnitude of the T cell response to the vaccine [114,130]. However, it should be noted that peptide vaccine is considered to be a very weak vaccine, generating less than 5% of clinical responses in a meta-analysis [131].

Preliminary clinical results appear to be more encouraging when anti-CTLA-4 was combined with DCs or live vector-based vaccines or recombinant live vectors.

In a phase I clinical trial on 16 patients with melanoma treated with the combination of MART-1 peptide-pulsed DCs and tremelimumab (anti-CTLA-4), a higher rate of durable objective tumor responses was observed than the rates expected with each agent administered alone [132].

In a phase II study of autologous monocyte-derived mRNA electroporated DCs (TriMixDC-MEL) plus ipilimumab in patients with pretreated advanced melanoma, the 6-month disease control rate was 51%, and the overall tumor response rate was 38% (including eight complete and seven partial responses) [133]. The observed antitumor activity compared favorably with the overall response rate (ORR) observed with ipilimumab monotherapy in numerous prospective clinical trials in patients with pretreated advanced melanoma [128,134,135].

Thirty metastatic castration-resistant prostate cancer patients were treated with a fixed dose of a poxviral vaccine (PROSTVAC) targeting prostate-specific antigen and escalating doses of ipilimumab administered after the vaccine. Among the 24 chemotherapy-naïve patients, 58% obtained a PSA decline with a ≥50% decline in six cases [136]. The median OS in the trial was 31.6 months, which compared favorably with the OS of 25.1 months observed in the phase II study using the PROSTVAC cancer vaccine alone in the same patient population [137,138].

Very few clinical trials combining cancer vaccine and PD-1-PD-L1 blockade have been reported. Seven patients with metastatic pancreatic cancer included in a pilot study received treatment with DC-based vaccine and nivolumab given one day before the vaccine. Using RECIST criteria, two partial responses were observed with OS after onset of therapy of 13 months and 5 months, respectively [139].

A multipeptide vaccine (gp100, MART-1, and NY-ESO-1 with Montanide ISA51) combined with nivolumab at various dosages (1 mg/kg, 3 mg/kg, or 10 mg/kg I.V.) was administered as adjuvant therapy in 33 resected stage IIIC and IV HLA-A2 melanoma patients [140]. An increase of antigen-specific T cells was observed after therapy, but the absence of difference between the various cohorts receiving different doses of nivolumab did not support a synergistic action of combined therapy [140].

The estimated median recurrence-free survival was 47.1 months and median OS was not reached, which compared favorably with historical control cohorts.

## 6. Conclusions and Perspectives

Preclinical data strongly support the combination of cancer vaccines and checkpoint inhibitor blockade or agonistic costimulatory antibodies in contrast with the rare and fairly negative results available in humans in terms of clinical benefit, especially when peptide vaccines were combined with ipilimumab. As reported in mice, the synergy between vaccines and immunomodulators may depend on the type of cancer vaccine [52]. In preclinical models, blockade of these cells or molecules synergized with HPV vaccine, but it was not always the same antagonist or agonist of costimulatory molecules that optimally synergized with selected HPV vaccines [31,97,141]. In this context, clinical results with more complex vaccines (DC-based vaccine or recombinant live vectors) appear to be more promising [133,137,139].

Few criteria have been proposed concerning the selection of inhibitory pathways or stimulatory pathways to be engaged with the cancer vaccine. Anti-CTLA-4 would more effectively inhibit T cells with higher avidity for antigen [142], while PD-1 blockade would more effectively inhibit T cells with lower avidity submitted to weak TCR activation [143,144]. Depending on the strength and potency of the vaccine, CTLA-4 or PD-1 signaling blockade may be preferred [26]. In mice, one study compared a peptide vaccine with various inhibitory or stimulatory antibodies and found that anti-4-1BB was superior to other antibodies when combined with peptide vaccine [97].

The timing of combination therapy is likely a critical point that is rarely addressed in clinical trials. In a murine model, anti-CTLA-4 mAb improved the antitumor effects of a DNA vaccine only when given with the second vaccine (boost) [145]. Similarly, in a prostate cancer model (Pro-TRAMP), Wada et al. showed an optimal response when anti-CTLA-4 was administered the same day or after vaccination [55]. Other studies have also reported that anti-CTLA-4 mAb is more efficient to improve the efficacy of cancer vaccines when given at the time of the boost rather than at primary immunization [145]. For agonist antibodies, such as anti-CD40, the best synergy was also observed when the antibody was administered at the time of the boost vaccination [61]. A better knowledge of the kinetics of appearance of inhibitory and activating receptors during the course of immunization should allow more adapted timing of combination therapy. In a model of lung adenocarcinoma, Koyama showed upregulation of alternative immune checkpoints, notably Tim-3, in mice with progressive tumors following anti-PD1 therapy. Sequential administration of anti-Tim-3 in anti-PD1-resistant tumors demonstrated clinical efficacy with enhanced survival of mice [146].

Despite the enthusiasm generated by the clinical success of checkpoint inhibitor blockade, more than 70% of cancer patients remain resistant to these treatments. One hypothesis is that only tumors with a high mutational load elicit a spontaneous T cell response and will be sensitive to reversal of anergy by PD-1-PD-L1/2 blockade. In a preclinical model of leukemia, it has been elegantly demonstrated that cancer vaccines cooperated with checkpoint blockade and allowed for immune control of cancers with low nonsynonymous mutation loads [147].

A better understanding of the discordance between the positive results obtained this combination in preclinical models and human studies, a better design of the timing of this combination, together with the selection of more powerful cancer vaccines would represent a prerequisite for successful combination therapy. Second-generation cancer vaccines should integrate this combination and would be indicated in patients resistant to checkpoint inhibitor blockade with no significant intratumoral CD8^+^ T cell infiltration [53,56].

## Figures and Tables

**Table 1:** Some examples of synergy between checkpoint inhibitor blockade or positive costimulatory pathway signaling and cancer vaccines in preclinical models.

Type of Checkpoint Inhibitor Blocked	Type of Tumor	Type of Vaccine	Clinical Benefit and Toxicity	Ref.
Anti-CTLA-4	SM1 cell line (mammary tumor)	1 × 10^6^ irradiated GM-CSF transduced SM1 cells (s.c.)	Complete regression of tumor volume and enhanced survival of mice	[52]
	B16-BL6 cell line (melanoma)	1 × 10^6^ irradiated GM-CSF–producing B16-BL6 and B16-F10 (sc)	Eradication of established tumors in 80% (68/85) of the cases. Development of depigmentation, starting at the site of vaccination or challenge and in most cases progressing to distant sites.	[53]
	TRAMP-C2 cell line (prostate cancer)	1 × 10^6^ cells irradiated GMTRAMP-C1/C2	Reduction in tumor incidence and tumor grade. Development of prostatitis accompanied by destruction of glandular epithelium of the male reproductive tract	[54]
	SP1 cell line (Prostate cancer)	GVAX: 1 × 10^6^ TRMPC-2HA admixed with 5 × 10^4^ B78H1-GM then irradiated	Dramatic increase in effector CD8^+^T cells in the prostate gland, and enhanced tumor-antigen directed lytic function and decreased tumor burden and histologic grade in ProHA × TRAMP mice	[55]
	B16/BL6 (melanoma TRAMP-C2 cell line (prostate cancer)	Fl3vax: irradiated 1 × 10^6^ Flt3L-expressing B16/BL6 (i.d.)	Rejection of established TRAMP prostate adenocarcinomas. Significantly greater prevention of the outgrowth of 5-day implanted B16-BL6 tumors than Gvax	[56]
	B16 cell line (Melanoma)	Synthetic peptide+ CpG-ODN adjuvant	Increased survival of mice with poorly immunogenic B16 melanoma in a therapeutic protocol, but ineffective in the prophylactic mode.	[57]
	11A-1 & MC-38 cell line (mammary and colon carcinoma	Modified vaccinia Ankara-expressing murine p53 +CpG-ODN	The combination of CpG ODN and CTLA-4 blockade synergized for the rejection of palpable 11A-1 and MC-38 tumors	[58]
	EG7-OVA cells (Thymoma)	2 × 10^6^ SIINFEKL-pulsed or unpulsed DCs (i.d.)	Rejection or slowed tumor growth in more than 60% of mice.	[59]
	CT26 cells (Colon cancer)	10^6^ cells peptide pulsed DCs (i.d.)	Increased mean survival of mice.	[60]
	MC-38 (Colon cancer)	2–3 × 10^5^ cells peptide pulsed DCs (i.d.)	Improvement of tumor-free survival of mice Immunized mice appeared healthy and maintained normal weight compared to mice mock-vaccinated with PBS.	[61]
	Meth A cells (Sarcoma)	Modified vaccinia Ankara (MVA) poxvirus encoding mutated p53 protein vaccine (i.p.)	Improvement of tumor-free survival (11/14) of mice receiving combination therapy	[62]
	MC-38 cells expressing CEA (Colon cancer)	1 × 10^7^ recombinant vaccinia vector carrying the genes for CEA, B7.1, ICAM-1, and LFA-3	Reduction of tumor volume complete eradication of the tumor (4 out of 20 mice). No signs of autoimmunity	[63]
Anti-CTLA-4 + anti-PD-1	B16-BL6 cells (Melanoma)	1 × 10^6^ B16 melanoma cells expressing either GM-CSF (Gvax) or Flt3-ligand (Fvax)	Reduction of tumor volume enhanced survival (75% survival with combined regimen versus 25% with monotherapy)	[64]
	CT26 cell line & ID8-VEGF cell line (colon and ovarian cancer)	1 × 10^6^ irradiated (150Gy) CT26-GVAX or ID8-VEGF-GVAX	Reduction of tumor volume and increased tumor rejection (between 75%–100% of tumor-free mice with combined therapy versus 30%–50% with monotherapy)	[65]
	B16-OVA cell line (melanoma)	Bacterial vaccine: The leucine–arginine auxotrophic S. Typhimurium A1-R strain 2 x 107 colony formation units	Eradication of established tumor in 80% of mice receiving the combined regimen	[66]
anti-CTLA-4 + anti-OX40	TUBO cell line (Breast cancer)	Anti–DEC-205/HER2 monoclonal antibodies	4-fold reduction of tumor volume and survival enhancement (40% survival versus 0%)	[67]
Anti-PDL-1	ID8 cell line (i.p.) (Ovarian cancer)	10^6^ irradiated ID8 cells expressing murine GM-CSF (ID8-GVAX) or Flt3-ligand (ID8-FVAX)	Rejection of ID8 tumor in 75% of tumor bearing mice increased proliferation and function of tumor antigen CD8^+^T cells	[65]
	MDA-MB-231 & MDA-MB-435 (breast cancer)	DC vaccine	Reduction of tumor volume and enhanced survival (60% of mice survived in the groups treated with DC vaccines versus 100% mortality with vaccine alone or anti-PDL1 alone)	[68]
anti-PD-1	B16F10, EL4, RMA-S cells (melanoma, thymoma, lymphoma)	DC vaccine+Poly-IC+anti-CD40 (Trivax)	Eradication of established tumor enhanced survival	[61]
	B16 cell line (melanoma)	A tumor cell based vaccine to create TLR agonists (TEGVAX)	Reduction of tumor volume (50% regression) appearance of vitiligo after treatment	[69]
	TC-1 cell line (lung cancer)	vaccine (E7/GM-CSF/anti-CD40)	Reduction of tumor volume (10-fold reduction) and enhanced survival (50%)	[70]
CD27 agonist + anti-PD-1	TC-1 cell line (lung cancer)	HELP-E7SH & E7SH DNA vaccines	Eradication of established tumor, resulting in 100% survival of the mice treated with combined therapy	[71]
Anti-BTLA	Transgenic mouse model mimicking thyroid adenocarcinoma	E7 expressed by herpes simplex virus (HSV)-1 glycoprotein D (gD)	Slowing of tumor progression resulting in 50% reduction of thyroid weight	[72]
VISTA blockade	B16-BL6 cells (melanoma)	Peptide-based cancer vaccine with TLR agonists as adjuvants	Eradication of established tumor protection against tumor rechallenge in 50% of mice	[73]
